# Safety and antiemetic efficacy of weekly administration of netupitant/palonosetron plus dexamethasone during 5 weeks of concomitant chemo-radiotherapy—the DANGER-emesis study

**DOI:** 10.1007/s00520-025-09573-9

**Published:** 2025-05-28

**Authors:** Sofie S. Detlefsen, Ditte S. Andersen, Anja Ø. Knudsen, Trine J. Nøttrup, Sören Möller, Gitte-Bettina Nyvang, Trine L. Jørgensen, Jørn Herrstedt, Christina H. Ruhlmann

**Affiliations:** 1 Department of Oncology, Odense University Hospital, Odense, Denmark; 2 Department of Clinical Research, University of Southern Denmark, Odense, Denmark; 3 Department of Oncology, Rigshospitalet, Copenhagen University Hospital, Copenhagen, Denmark; 4 OPEN - Open Patient Data Explorative Network, Department of Clinical Research, University of Southern Denmark, Odense, Denmark; 5 Department of Clinical Oncology, Zealand University Hospital, Roskilde, Denmark; 6 University of Copenhagen, Copenhagen, Denmark

**Keywords:** Concomitant chemoradiotherapy, Nausea, NEPA, Netupitant, Palonosetron, Vomiting

## Abstract

**Purpose:**

Netupitant 300 mg/palonosetron 0.5 mg (NEPA) would be ideal as antiemetic prophylaxis for patients receiving weekly cisplatin, as it would reduce concurrent medication intake compared to the 3-day aprepitant regimen. However, due to the longer half-life of netupitant (~ 88 h), weekly administration could potentially lead to accumulation and toxicity. This study aims to investigate the safety and antiemetic efficacy of weekly administration of NEPA plus dexamethasone (DEX) in patients treated for cervical cancer with radiotherapy and weekly cisplatin 40 mg/m^2^.

**Methods:**

This single-arm, open-label, phase II study evaluated patients with cervical cancer receiving NEPA and DEX before weekly cisplatin and concomitant radiotherapy for up to 5 weeks. Safety was assessed during weekly adverse event (AE) assessments. Efficacy was evaluated using Patient Diaries reporting daily nausea, vomiting, and use of rescue medication during the study period.

**Results:**

Between October 8, 2018, and January 2, 2024, 73 patients were recruited from two Danish departments of oncology; 37 completed all five weekly cycles. The majority of AEs were of mild or moderate intensity, with fatigue being the most frequently observed (95% of patients). Seven (10%) patients encountered ≥ 1 grade 3 treatment-related AEs (TRAEs). No grade 4 TRAEs or deaths were observed. In terms of efficacy, no vomiting and no nausea days 1–35 were 86% and 18%, respectively. Mean time to first emetic episode was 9 days.

**Conclusion:**

Weekly NEPA administration was safe, well-tolerated, and highly effective during concomitant radiotherapy and weekly cisplatin.

**Trial registration:**

This trial is registered at ClinicalTrials.gov (NCT03668639-2018–09-10).

**Supplementary Information:**

The online version contains supplementary material available at 10.1007/s00520-025-09573-9.

## Introduction

Nausea and vomiting are some of the most distressing, yet common and often undertreated symptoms for patients with cancer undergoing radiotherapy or concomitant chemo-radiotherapy [1]. This can lead to a significant negative impact on quality of life and the ability to perform daily activities, potentially leading to non-completion or delay of the chemo-radiotherapy [2, 3].

Among patient-related risk factors for chemotherapy-induced nausea and vomiting (CINV) are age and gender, with young women having a higher risk of developing severe emetic side effects [4]. In contrast, the risk factors associated with radiotherapy are less well defined. Only two significant radiotherapy-related risk factors have been verified: the irradiated organs (upper abdomen) and field size (> 400 cm^2^) [5]. Furthermore, it remains unclear whether administering radiotherapy concomitantly with chemotherapy will result in an additive or synergistic effect on nausea and vomiting [6]. A study found that, despite the use of antiemetic prophylaxis, patients undergoing radiotherapy and concomitant weekly cisplatin ranked nausea as the fifth most severe symptom during treatment [7].

This challenge was further explored by Ruhlmann et al. in the GAND-emesis trial (GAND-emesis), which investigated the neurokinin-1 (NK_1_) receptor antagonist (RA) fosaprepitant in women with cervical cancer undergoing fractionated radiotherapy and concomitant weekly cisplatin. The results demonstrated that adding weekly fosaprepitant (single dose of 150 mg i.v.) to a regimen of palonosetron (single dose of 0.25 mg i.v.) and dexamethasone (DEX) increased the probability of completing 5 weeks of treatment without emesis by 17% compared to placebo [8].

The highly emetogenic chemotherapeutic drug, cisplatin, causes nausea and vomiting by increasing levels of serotonin and substance P, which binds to 5-hydroxytryptamine-3 (5-HT_3_) and NK_1_ receptors, respectively. Activation of these receptors is the main actor in initiating the pathway of CINV [9]. The current antiemetic guidelines by the Multinational Association of Supportive Care in Cancer (MASCC) and the European Society for Medical Oncology (ESMO) recommend a three-drug regimen, including a 5-HT_3_-RA, dexamethasone, and the NK_1_-RA, aprepitant/fosaprepitant, to prevent acute and delayed nausea and vomiting following radiotherapy and concomitant weekly cisplatin [1]. Aprepitant is administered orally on days 1 to 3, while fosaprepitant, an intravenous prodrug of aprepitant, is administered on day 1 only, both on a weekly basis [10, 11]. Other NK_1_-RAs, such as netupitant and rolapitant, differ from aprepitant in terms of their plasma half-lives: aprepitant has a plasma half-life of 9 to 13 h, while netupitant’s is 88 h and rolapitant’s is approximately 180 h [12, 13]. Notably, the extended half-life of netupitant, combined with its high receptor occupancy, results in a prolonged NK_1_ receptor blockade, thereby probably extending its therapeutic effect compared to aprepitant, but also with a risk of cumulative adverse effects [14].

A fixed-dose combination of netupitant 300 mg and palonosetron 0.5 mg (NEPA) is available for oral use administered prior to emetogenic chemotherapy. The plasma half-life of netupitant is about eight times the plasma half-life of aprepitant. Weekly administration of NEPA has not been assessed for safety and is currently not recommended during weekly cisplatin and concurrent radiotherapy [1]. It is also unknown whether a weekly single dose of NEPA and dexamethasone enhances compliance and improves antiemetic efficacy compared to antiemetic prophylaxis with aprepitant/fosaprepitant, palonosetron, and dexamethasone.

The aim of this prospective cohort study was to investigate the safety and antiemetic efficacy of NEPA in combination with dexamethasone during weekly administration in women with cervical cancer, scheduled to receive fractionated radiotherapy and concomitant weekly cisplatin 40 mg/m^2^ for 5 weeks. The efficacy was compared to results from a previous randomised trial including fosaprepitant [8].

## Methods

### Study design and participants

This prospective study was a multicentre, single-arm, open-label, phase II study. Patients were recruited from two departments of oncology in Denmark. Eligible patients were ≥ 18 years old with a diagnosis of cervical cancer, scheduled to receive fractionated external beam radiotherapy (1.8–2.0 Gy/fraction) and concomitant weekly cisplatin 40 mg/m^2^, maximum 70 mg, for 5 weeks. If brachytherapy was indicated as part of the primary cancer treatment, it should be executed after the third cycle of weekly cisplatin. Patients were chemo- and radiotherapy-naïve, had an Eastern Cooperative Oncology Group (ECOG) performance status ≤ 2, and had adequate haematologic and metabolic status (details specified in the protocol) allowing for weekly cisplatin. Furthermore, patients were required to read and understand Danish and to complete questionnaires and daily records in a Patient Diary throughout the study period.

Patients were excluded if they were pregnant, had a concurrent malignant diagnosis (except non-melanoma skin cancers), or any medical condition predisposing to nausea or emesis. Exclusion also applied to those with clinically significant nausea (moderate or severe) or emesis 24 h before the first dose of study medication or those who used high or moderate emetogenic medications within 48 h before study initiation. Patients on stable opiate doses without emetogenic events in the 24 h preceding the first dose of the study drug were eligible. Moderate CYP-3 A4 inhibitors or inducers were prohibited within 7 and 30 days, respectively, prior to study initiation.

The trial was registered with clinicaltrials.gov, number NCT03668639.

A signed, written informed consent was obtained prior to any study procedures or assessments being initiated. The study was approved by the Ethics Committee (Acadre number: 17/35157) and the Danish Medicines Agency (EudraCT number: 2017–004031-37). It was conducted in accordance with the principles of Good Clinical Practice, as well as all applicable patient privacy requirements in Denmark (protection by the “Act on Processing of Personal Data” and the “Health Care Act”), and the guiding principles of the Declaration of Helsinki. Data were entered into a logged, web-based REDCap database hosted by Open Patient data Explorative Network (OPEN), Odense, Denmark.

### Procedures

Patients received fractionated radiotherapy and concomitant cisplatin weekly for five cycles (cycle defined as the 7-day period between cisplatin treatments). Patient eligibility and continued adherence to the entry criteria, including weekly blood samples (i.e. haematology, creatinine, and liver enzymes) were reviewed by investigators before each cycle.

Subjects were administered a single dose of oral NEPA and 12 mg of oral DEX, 60 and 30 min, respectively, before cisplatin on study day 1. They were dispensed tablets of oral DEX for self-administration on study days 2–4 (8 mg on days 2–3, 4 mg on day 4). Rescue antiemetic medication was allowed to relieve symptoms of emergent nausea and/or vomiting.

From study day 1 in the first cycle, each patient completed a 7-day validated Patient Diary for up to 5 weeks, documenting daily episodes of emesis (defined as one or more consecutive vomits or dry retches (an attempt of vomiting without emptying) separated by at least 1 min), nausea, rescue medication use (i.e. drug name, dose, and number of tablets), and whether DEX was taken on days 2–4 as prescribed. Severity of nausea was graded as either none, mild, moderate, or severe. The diaries were returned on study day 1 each week, with Patient Diary cycle 5 collected at the follow-up visit, scheduled 7–14 days after study day 1 of the final cycle. Investigators reviewed the Patient Diary with the patient to ensure correct completion.

Investigators evaluated a list of 34 items representing potential adverse events (AEs) and their relation to the study drug at baseline (prior to study initiation), weekly throughout the study, and at the follow-up visit. AEs were considered treatment-related adverse events (TRAEs) if the relationship to NEPA or NEPA/DEX was deemed as definitely, probably or possibly related, or if it was missing. The severity of AEs was graded in concordance with the National Cancer Institute Common Terminology Criteria of Adverse Events (NCI CTCAE) version 5.0 [15]. The investigator was responsible for the detection of events meeting the criteria of an AE and serious adverse events (SAEs). The follow-up period was defined as the period from the first study drug dose to the last AE evaluation.

### Outcomes

The first co-primary objective was to explore the safety of the antiemetic regimen NEPA and DEX during five weeks of fractionated (5 days a week) radiotherapy and concomitant weekly cisplatin at a dose of 40 mg/m^2^, maximum 70 mg. The second co-primary objective aimed to investigate NEPA and DEX in terms of the proportion of subjects with no vomiting (i.e. sustained no emesis rate) during the treatment period.

The secondary objectives were to investigate NEPA and DEX in terms of the proportion of subjects achieving complete response (no vomits, no dry retches, and no need for rescue medication), as well as no vomiting, no significant nausea (none or mild nausea), and no nausea within the first 5 and the 35 days following the initiation of chemo-radiotherapy. The final secondary objective was to investigate NEPA and DEX in terms of time to the first emetic episode. Exploratory analysis comparing efficacy of a historical cohort using fosaprepitant, palonosetron, and DEX (GAND-emesis) to the DANGER-emesis antiemetic regimen was planned.

### Missing data

AE registrations that were missing were left as such. Entire Patient Diaries that were missing were handled by using the investigator-assessed AE registration, which included information on nausea and vomiting and the corresponding time frame. If only partial registrations regarding nausea, emetic episodes, and the use of rescue medication in a Patient Diary were answered, the missings were replaced with the most severe experienced outcome in that specific diary. If the worst experienced incidence was no nausea or emesis, the AE registration was used to register instances of nausea and/or vomiting in that specific period. Patients who went off radio- or chemotherapy were censored from further analyses.

### Statistical analyses

Outcomes regarding safety were assessed using descriptive analyses on the intention-to-treat (ITT) population, consisting of all patients who received at least one dose of study medication and had at least one AE registration after treatment administration.

The co-primary outcome regarding efficacy was described using a Kaplan–Meier plot showing the cumulative incidence of patients (ITT population) who sustained no emesis after initiating chemo-radiotherapy.

The secondary efficacy outcomes regarding the proportion of subjects with complete response, no vomiting, no significant nausea, and no nausea were retrospectively compared with data from the GAND-emesis study (fosaprepitant group) using Fisher’s exact test. The outcome concerning the mean time to first emetic episode was compared with GAND-emesis, as well, using a two-sample *t*-test. A significance level of 0.05 was used. Secondary efficacy data were analysed based on both the ITT and per-protocol (PP) cohorts.

Analyses were performed using Stata software (version 18).

## Results

**Fig. 1:**
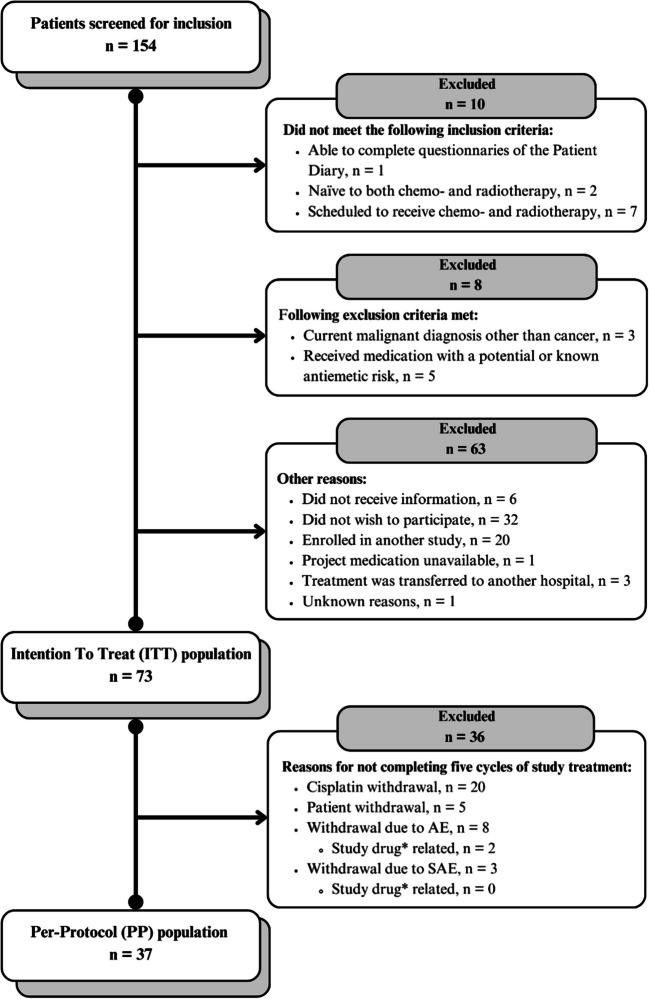
Flowchart of patient enrolment. An asterisk (*) symbol means it is related to NEPA

From October 8, 2018, to January 2, 2024, 154 patients were screened, and 73 patients were eligible and received at least one dose of the study medication, constituting the ITT-population (Fig. 1). Table 1 shows the characteristics of the study population (ITT) compared to the historical GAND-emesis fosaprepitant cohort. The PP-population consisted of 37 patients who successfully completed all five cycles of chemo-radiotherapy, including study medication.

**Table 1:** Baseline demographics and clinical characteristics. The table displays the actual DANGER-emesis cohort compared to the historical GAND-emesis cohort

	DANGER-emesis, *n* = 73	GAND-emesis, fosaprepitant group, *n* = 118
Age, years (median; range)	47 (range 23–82)	48 (range 25–74)
ECOG performance status (*n*; %)		
0	53 (73%)	102 (86%)
1	18 (25%)	16 (14%)
2	2 (3%)	0 (0%)
External beam radiation (median; range)		
Total dose external beam radiation therapy (tumour; Gy)	45 (range 45–50)	50 (range 50–50)
Total dose (elective lymph nodes; Gy)	45 (range 45–55)	45 (range 45–49)
Brachytherapy (*n*; %)	56 (77%)	88 (75%)
Timing of brachytherapy (*n*; %)		
After 3 cycles	0 (0%)	11 (13%)
After 4 cycles	15 (26%)	10 (11%)
After 5 cycles	43 (74%)	67 (76%)
Extension of radiotherapy field (*n*; %)		
Above the promontory	65 (89%)	39 (33%)
Below the promontory	8 (11%)	79 (67%)
Cisplatin 40 mg/m^2^ (*n*; %)	73 (100%)	118 (100%)
Alcoholic drinks per week (*n*; %)		
0–10	65 (90%)	111 (94%)
> 10	7 (10%)	7 (6%)
Cycles of chemo-radiotherapy completed (*n*; %)		
Cycle 1	73 (100%)	118 (100%)
Cycle 2	66 (90%)	100 (85%)
Cycle 3	59 (81%)	94 (80%)
Cycle 4	52 (71%)	81 (69%)
Cycle 5	37 (51%)	71 (60%)
Median follow-up period, days^a*^ (median; IQR)	30 (IQR 21–36)	35 (IQR 21–35)

*ECOG* Eastern Cooperative Oncology Group

^a*^Date of first dose of study drug until last AE-evaluation (days)

### Safety

Frequencies of AEs are presented in Table 2. At baseline, prior to treatment, medical history demonstrated that 75% of patients had at least one symptom. Throughout the study period, all patients experienced at least one AE, and 95% experienced at least one TRAE (Table 3). The majority of AEs and TRAEs were of mild or moderate intensity and did not accumulate over multiple cycles. The proportion of patients experiencing TRAEs decreased from 84% at the end of cycle 1 to 65% by the end of cycle 5.

**Table 2:** Overview of adverse events during the study. Adverse events (AEs), serious adverse events (SAEs), treatment-related AEs (TRAEs), and treatment-related SAEs (TRSAEs) shown per cycle and in total (*n*; %)

	Cycle						
AE type	Baseline, *n* = 73	Cycle 1, *n* = 73	Cycle 2, *n* = 66	Cycle 3, *n* = 59	Cycle 4, *n* = 52	Cycle 5, *n* = 37	Total, *n* = 73
≥ 1 AE	55 (75%)	72 (99%)	65 (98%)	59 (100%)	48 (92%)	32 (86%)	73 (100%)
≥ 1 AE grade 3	4 (5%)	3 (4%)	7 (11%)	7 (12%)	8 (15%)	9 (24%)	24 (33%)
≥ 1 AE grade 4	0 (0%)	0 (0%)	0 (0%)	0 (0%)	0 (0%)	1 (3%)	1 (1%)
≥ 1 SAE	0 (0%)	1 (1%)	3 (5%)	1 (2%)	1 (2%)	2 (5%)	5 (7%)
≥ 1 TRAE	-	61 (84%)	54 (82%)	51 (86%)	40 (77%)	24 (65%)	69 (95%)
≥ 1 TRAE grade 3	-	1 (1%)	5 (8%)	4 (7%)	3 (6%)	2 (5%)	7 (10%)
≥ 1 TRAE grade 4	-	0 (0%)	0 (0%)	0 (0%)	0 (0%)	0 (0%)	0 (0%)
≥ 1 TRSAE	-	0 (0%)	0 (0%)	0 (0%)	0 (0%)	0 (0%)	0 (0%)

**Table 3:** Treatment-related adverse events (TRAEs) in the ITT population. TRAEs occurring in ≥ 2% of patients are shown. No grade 4 or 5 TRAEs were observed. The column All grades represents the number of patients with grades 1, 2, and/or 3 TRAEs, counted once per patient regardless of the number of different grades experienced

	TRAEs (grade)			
TRAE (*n*; %)	Grade 1	Grade 2	Grade 3	All grades
Total	68 (93%)	44 (60%)	7 (10%)	69 (95%)
Abdominal pain	12 (16%)	2 (3%)	0 (0%)	13 (18%)
Cardiac disorders, other	2 (3%)	0 (0%)	0 (0%)	2 (3%)
Constipation	43 (59%)	12 (16%)	0 (0%)	49 (67%)
Decreased appetite	7 (10%)	2 (3%)	0 (0%)	7 (10%)
Diarrhoea	2 (3%)	1 (1%)	0 (0%)	3 (4%)
Dizziness	21 (29%)	3 (4%)	0 (0%)	22 (30%)
Dyspepsia	26 (36%)	9 (12%)	0 (0%)	28 (38%)
Eye disorders, other	3 (4%)	0 (0%)	0 (0%)	3 (4%)
Fatigue	10 (14%)	11 (15%)	2 (3%)	19 (26%)
Flatulence	10 (14%)	0 (0%)	0 (0%)	10 (14%)
Flushing	2 (3%)	0 (0%)	1 (1%)	2 (3%)
Gastrointestinal disorders, other	3 (4%)	0 (0%)	1 (1%)	4 (5%)
Headache	17 (23%)	1 (1%)	0 (0%)	17 (23%)
Hiccups	10 (14%)	0 (0%)	0 (0%)	10 (14%)
Insomnia	28 (38%)	16 (21%)	2 (3%)	36 (49%)
Liver transaminases increased	10 (14%)	1 (1%)	1 (1%)	11 (15%)
Nausea	2 (3%)	0 (0%)	0 (0%)	2 (0%)
Palmar-plantar erythrodysesthesia	4 (5%)	0 (0%)	0 (0%)	4 (5%)
Tremor	2 (3%)	0 (0%)	0 (0%)	2 (3%)
Urticaria	3 (4%)	1 (1%)	0 (0%)	4 (5%)

The most common TRAEs (occurring in > 20% of patients; see Table 3) were constipation (67%), dizziness (30%) dyspepsia (38%), fatigue (26%), and headache (23%). Among all 73 patients, 68 (93%) and 44 (60%) experienced grades 1 and 2 TRAEs, respectively. Seven (10%) patients experienced grade 3 TRAEs (i.e. abdominal distension, fatigue, flushing, increased liver transaminase, and insomnia, see Table 3), but none led to study discontinuation. No grade 4 TRAEs or treatment-related SAEs (TRSAE) were observed. Two (3%) patients discontinued the study due to AEs judged related to NEPA (urticaria grade 2 and fatigue grade 2, respectively), and one (1%) patient was withdrawn due to an AE related to DEX.

Among the severe AEs (grades 3 and 4), 24 (33%) patients experienced an AE grade 3, and one patient developed neutropenia grade 4 (Supplementary, Table S1). Five (7%) patients experienced ≥ 1 SAEs (Table 2). Three of the SAEs (diverticulitis, malaise, and urticaria) led to study discontinuation (Fig. 1). No deaths occurred during the study period.

### Efficacy

**Fig. 2:**
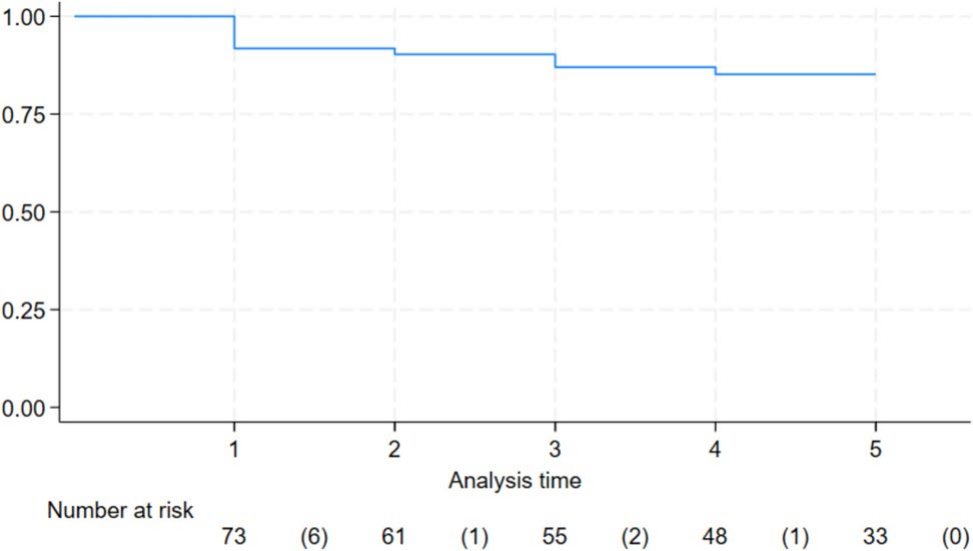
Kaplan–Meier plot illustrating the probability of patients with sustained no emesis over time (weeks since first dose of study treatment). The values listed beneath each week represent the total number of patients at risk during that specific week. The numbers in the parentheses indicate the number of patients experiencing emesis each week

The proportion of patients (ITT-population) with sustained no emesis 5 weeks after initiating chemo-radiotherapy was 86% (Fig. 2). The mean time to first emetic episode was 9 days (SD 9.3). When comparing retrospectively to the fosaprepitant group of the GAND-emesis study (11.25 days, SD 9), no significant difference was observed. While the efficacy outcomes days 1–5 did not show any difference when comparing with GAND-emesis (Table 4), a highly statistically significant difference in complete response (52% vs 24%) and no significant nausea (62% vs 26%) on days 1–35 (*p* < 0.001 and *p* < 0.001, respectively) in favour of the DANGER-emesis regimen was demonstrated. The use of rescue antiemetic drugs is shown in Table S2, Supplementary. Most commonly, domperidone was used (92% of cases).

**Table 4:** Secondary efficacy endpoints. Comparing efficacy endpoints between the DANGER-emesis cohort (ITT population) and the GAND-emesis three-drug experimental group. Asterisk (*) symbol indicates fosaprepitant group: fosaprepitant, palonosetron, and dexamethasone day 1 plus dexamethasone days 2–4 weekly. Data are *n* (%) or mean (SD). Significant results in bold

	Study cohort		
Efficacy endpoint	DANGER-emesis, *n* = 73	GAND-emesis, fosaprepitant group*, *n* = 118	*p* value
Complete response			
Days 1–5	56 (77%)	85 (72%)	0.503
Days 1–35	38 (52%)	28 (24%)	** < 0.001**
No vomiting			
Days 1–5	68 (93%)	107 (91%)	0.604
Days 1–35	63 (86%)	91 (77%)	0.135
No significant nausea			
Days 1–5	63 (86%)	95 (81%)	0.332
Days 1–35	45 (62%)	31 (26%)	** < 0.001**
No nausea			
Days 1–5	36 (49%)	55 (47%)	0.766
Days 1–35	13 (18%)	18 (15%)	0.689
Mean time to first emetic episode	9 (SD 9.30)	11.25 (SD 9.00)	0.099

When determining the efficacy outcomes for the PP population, a slightly less but still significant improvement was observed for complete response (46%, *p* = 0.013) and no significant nausea (57%, *p* = 0.001) during days 1–35 compared with GAND-emesis.

## Discussion

### Summary of main findings

To our knowledge, this is the first study to investigate safety and efficacy during weekly administration of NEPA and DEX prior to concomitant radiotherapy and weekly cisplatin. We found that weekly NEPA administration was well tolerated and did not raise any safety concerns, as 7 (10%) patients experienced grade 3 TRAEs, none of which led to study discontinuation, and no grade 4 TRAEs, TRSAEs, or deaths were reported. Furthermore, the study showed that weekly NEPA was highly effective, with 86% experiencing no vomiting during the 5-week study period. When compared retrospectively to the GAND-emesis cohort using the NK_1_-RA fosaprepitant, there was no statistically significant difference in efficacy for days 1–5 following chemo-radiotherapy. However, a statistically significant improvement in complete response and no significant nausea was observed over days 1–35, indicating that the main antiemetic improvement is to be seen over multiple cycles during concurrent radiotherapy.

### Safety

When comparing the TRAEs observed in this study with the described adverse reactions in the Summary of Product Characteristics of NEPA (Akynzeo®), no new TRAEs were identified when administered weekly, apart from palmar-plantar erythrodysesthesia, which may be associated with the chemotherapy rather than the antiemetics [13, 16]. The common adverse reactions (1–10%) reported for Akynzeo®, including headache, constipation, and fatigue, are specifically attributed to palonosetron and are therefore unlikely to be influenced by the extended plasma half-life of netupitant. The study revealed a higher frequency of TRAEs compared to those documented in the product information, which may be attributed to the extensive list of 34 symptoms that patients were asked about during each weekly appointment. Investigators might tend to mark an event as “possibly related” rather than “unlikely related” when uncertain or when dealing with AEs that are difficult to assess in relation to NEPA or NEPA/DEX, potentially inflating TRAE registrations. For example, the non-specific symptom of fatigue affected 95% of patients, with 26% considered treatment-related, of which 58% were deemed “possibly related” to NEPA. Notably, despite the use of weekly netupitant, with an extended plasma half-life, compared with the recommended NK_1_-RA aprepitant/fosaprepitant, the frequency of AEs and TRAEs did not increase over multiple cycles.

The possible accumulation of NEPA after multiple doses has been addressed in several studies. Haab et al. examined daily doses of netupitant (50 mg, 100 mg, 200 mg) for 8 weeks in patients with overactive bladder, while Spinelli et al. investigated a high single dose of NEPA (600 mg netupitant/1.5 mg palonosetron) in healthy subjects. Both studies reported a low frequency of TRAEs and no TRSAEs or deaths, supporting our findings that high or frequent doses of netupitant or NEPA are well tolerated without notable safety concerns [14, 17]. NEPA has also been investigated as antiemetic prophylaxis in patients undergoing haematopoietic stem cell transplantation. In these studies, using multiple-day highly emetogenic conditioning chemotherapy, two to three repeated doses of NEPA administered every 48 to 72 h were found safe and efficacious [18–20].

### Efficacy

When retrospectively comparing the efficacy outcomes of the ITT cohort with GAND-emesis using the NK_1_-RA prodrug fosaprepitant, no significant differences were observed on days 1–5. Looking at a longer follow-up period (days 1–35), no significant nausea was significantly improved (*p* < 0.001) which may have led to less need for rescue medication, as complete response was also significantly improved (*p* < 0.001) compared with GAND-emesis. Similar results apply to the PP-cohort, which consists of patients who completed all five cycles. This improvement may be due to netupitant’s longer plasma half-life compared to fosaprepitant. Additionally, netupitant maintains persistent NK_1_ receptor occupancy, reaching 90% after 2.2 h and gradually declining from 88.9% at 24 h to 59.6% after 10 days [21]. In contrast, receptor occupancy for aprepitant/fosaprepitant remains ≥ 98% within the first 48 h but decreases to below 60% after 5 days [22].

Although patients in both the DANGER-emesis and GAND-emesis studies received fractionated radiotherapy and concomitant weekly cisplatin, differences in the radiotherapy procedures (see Table 1) must be considered when interpreting the significantly improved long-term efficacy observed in this study. GAND-emesis patients received a higher median tumour-targeted radiation dose (50 Gy vs 45 Gy in DANGER-emesis), and 13% initiated brachytherapy after three cycles (0% in DANGER-emesis). Conversely, more DANGER-emesis patients had their radiotherapy field extended above the promontory.

Despite the high antiemetic efficacy observed in this study, some patients still experienced significant nausea (38%) and vomiting (14%) after 35 days of treatment. Further research is needed to identify patients who may benefit from adding a fourth antiemetic agent, specifically olanzapine, to the existing 3-drug regimen for more effective symptom management [23]. Undoubtedly, the fractionated radiotherapy contributes to the overall emetogenicity of concurrent chemo-radiotherapy. Based on dose plans from the GAND-emesis study, a dosimetric study to identify predictors for vomiting during chemo-radiation was presented as a poster at ESMO Congress 2024. The study showed that the risk of vomiting is significantly higher when the bowel volume that receives 15 Gy or more (V_15_) is above 1000 cm^3^ compared to V_15_ below 1000 cm^3^ [24]. This predictor could be used to stratify for patients benefitting from the add-on of olanzapine to the antiemetic prophylaxis. The full publication is pending.

### Limitations

A limitation concerning efficacy outcomes is that only 37 of 73 patients (51%) completed all five weekly antiemetic therapies compared to 60% in the GAND-emesis study (fosaprepitant arm of the study). Additionally, not all patients adequately completed or returned the Patient Diaries, possibly due to incomplete instructions, worsened general condition, or nausea and vomiting. To address this, missing data were replaced with the worst observed outcome within the same diary, potentially enhancing the credibility of the efficacy results.

General limitations of this study include the uncertainty of reproducibility of the safety of weekly administration of NEPA in men, as only women were tested, although there is no reason to assume that men would tolerate NEPA differently. For example, both men and women with head and neck cancer undergoing weekly chemo-radiotherapy may also benefit from weekly NEPA [25]. Additionally, the inclusion period was prolonged due to unforeseen competitive studies and fewer newly diagnosed patients with cervical cancer, possibly due to the effect of the HPV-vaccine programme.

Inherently, the efficacy analysis between the historical GAND-emesis cohort and the DANGER-emesis cohort represents a major limitation and should be interpreted with caution. Another important limitation for the interpretation of the efficacy comparison data is that the DANGER-emesis study was not powered for the secondary outcomes, i.e. comparing efficacy with the GAND-emesis efficacy data.

### Perspectives

From a clinical perspective, the fixed-dose combination of NEPA may, compared to a 3-day aprepitant regimen, simplify the treatment regimen for patients undergoing chemo-radiotherapy, requiring only a single weekly tablet of NEPA regimen. This could improve patient compliance and, in this regard, have similar advantages as the day 1 administration of fosaprepitant compared to days 1–3 administration of aprepitant. The superiority of NEPA compared to fosaprepitant plus palonosetron in the current treatment setting remains unsettled. Both regimens still necessitate DEX self-administration on days 1–4, and it is possible that the DEX dose and duration could be reduced while still maintaining optimal antiemetic efficacy. Future studies are needed to determine the optimal DEX dosage.

Currently, no specific 5-HT_3_-RA is preferred according to the MASCC/ESMO antiemetic guidelines for patients receiving weekly cisplatin and concomitant radiotherapy. However, in both the GAND- and DANGER-emesis studies, patients were administered palonosetron. A pilot study by Ganesh et al. suggests that palonosetron significantly improved the control of radiotherapy-induced nausea and vomiting compared to a historical cohort using ondansetron. Further prospective studies are needed to investigate if palonosetron should be the preferred 5-HT_3_-RA in radiotherapy- and chemo-radiotherapy regimens [26].

## Conclusion

In conclusion, NEPA was well tolerated among patients with cervical cancer receiving fractionated radiotherapy and concomitant weekly cisplatin, as no TRSAEs or deaths were observed during the study, as well as a generally low frequency of severe TRAEs. The results of this study have the potential to update the MASCC/ESMO antiemetic guidelines recommendation for prophylaxis during weekly cisplatin and concurrent radiotherapy by adding netupitant as an optional NK_1_-RA parallel to fosaprepitant.

## Supplementary Information

Below is the link to the electronic supplementary material.

Supplementary file1 (DOCX 22 KB)

## Data Availability

The data that support the findings of this study are available upon reasonable request to the corresponding author.
