# Supplementary material for: Safety and antiemetic efficacy of weekly administration of netupitant/palonosetron plus dexamethasone during 5 weeks of concomitant chemo-radiotherapy—the DANGER-emesis study
Source: Support Care Cancer. 2025 May 28;33(6):509. doi: 10.1007/s00520-025-09573-9 (PMC12119652; doi:10.1007/s00520-025-09573-9)
Supplement: Supplementary file 1 — Supplementary file1 (DOCX 22 KB) [file 520_2025_9573_MOESM1_ESM.docx]

# Supplementary

# *Safety and antiemetic efficacy of weekly administration of netupitant/palonosetron plus dexamethasone during five weeks of concomitant chemo-radiotherapy – the DANGER-emesis Study*

## Table S1

Legend Table S1A: Summary of Adverse Events for each cycle

Text Table S1A: Data are n (%)

| AEs | Baseline  n = 73 | Cycle 1  n = 73 | Cycle 2  n = 66 | Cycle 3  n = 59 | Cycle 4  n = 52 | Cycle 5  n = 37 |
| --- | --- | --- | --- | --- | --- | --- |
| Abdominal pain | 19 (26%) | 28 (38%) | 26 (39%) | 26 (44%) | 20 (38%) | 13 (35%) |
| Alopecia | 1 (1%) | 2 (3%) | 4 (6%) | 5 (8%) | 4 (8%) | 3 (8%) |
| Blood creatinine increased | 2 (3%) | 1 (1%) | 1 (2%) | 0 (0%) | 0 (0%) | 0 (0%) |
| Cardiac disorders, other | 1 (1%) | 1 (1%) | 4 (6%) | 2 (3%) | 2 (4%) | 3 (8%) |
| Constipation | 21 (29%) | 48 (66%) | 36 (55%) | 24 (41%) | 12 (23%) | 6 (16%) |
| Decreased appetite | 12 (16%) | 29 (40%) | 21 (32%) | 20 (34%) | 14 (27%) | 11 (30%) |
| Diarrhoea | 4 (5%) | 6 (8%) | 14 (21%) | 20 (34%) | 15 (29%) | 15 (41%) |
| Dizziness | 7 (10%) | 21 (29%) | 21 (32%) | 16 (27%) | 13 (25%) | 6 (16%) |
| Dyspepsia | 7 (10%) | 24 (33%) | 25 (38%) | 27 (46%) | 24 (46%) | 12 (32%) |
| Eye disorders, other | 2 (3%) | 5 (7%) | 6 (9%) | 7 (12%) | 5 (10%) | 1 (3%) |
| Fatigue | 36 (49%) | 59 (81%) | 56 (85%) | 49 (83%) | 43 (83%) | 28 (76%) |
| Flatulence | 14 (19%) | 19 (26%) | 32 (48%) | 39 (66%) | 32 (62%) | 18 (49%) |
| Gastrointestinal disorders, other | 1 (1%) | 1 (1%) | 0 (0%) | 1 (2%) | 4 (8%) | 0 (0%) |
| General disorders, other | 1 (1%) | 2 (3%) | 1 (2%) | 3 (5%) | 3 (6%) | 3 (8%) |
| Headache | 6 (8%) | 18 (25%) | 13 (20%) | 11 (19%) | 10 (19%) | 9 (24%) |
| Hematologic disorders, other | 0 (0%) | 1 (1%) | 1 (2%) | 3 (5%) | 4 (8%) | 4 (11%) |
| Hiccups | 0 (0%) | 8 (11%) | 4 (6%) | 3 (5%) | 2 (4%) | 1 (3%) |
| Infectious disorders, other | 2 (3%) | 3 (4%) | 3 (5%) | 2 (3%) | 3 (6%) | 0 (0%) |
| Insomnia | 19 (26%) | 28 (38%) | 24 (36%) | 22 (37%) | 23 (44%) | 17 (46%) |
| Investigations, other | 2 (3%) | 4 (5%) | 6 (9%) | 6 (10%) | 5 (10%) | 1 (3%) |
| Liver transaminases increased | 2 (3%) | 3 (4%) | 8 (12%) | 11 (19%) | 3 (6%) | 1 (3%) |
| Muscular disorders, other | 7 (10%) | 10 (14%) | 8 (12%) | 6 (10%) | 5 (10%) | 6 (16%) |
| Nausea | 3 (4%) | 25 (34%) | 23 (35%) | 20 (34%) | 15 (29%) | 14 (38%) |
| Nervous disorders, other | 2 (3%) | 3 (4%) | 3 (5%) | 2 (3%) | 1 (2%) | 1 (3%) |
| Neutropenia | 0 (0%) | 0 (0%) | 0 (0%) | 1 (2%) | 3 (6%) | 6 (16%) |
| Palmar plantar erythrodysesthesia | 3 (4%) | 3 (4%) | 3 (5%) | 4 (7%) | 5 (10%) | 1 (3%) |
| Psychological disorders, other | 1 (1%) | 2 (3%) | 1 (2%) | 1 (2%) | 1 (2%) | 1 (3%) |
| Respiratory disorders, other | 3 (4%) | 4 (5%) | 4 (6%) | 7 (12%) | 4 (8%) | 4 (11%) |
| Sensory neuropathy | 7 (10%) | 15 (21%) | 18 (27%) | 15 (25%) | 9 (17%) | 6 (16%) |
| Skin disorders, other | 3 (4%) | 5 (7%) | 4 (6%) | 4 (7%) | 5 (10%) | 1 (3%) |
| Urticaria | 2 (3%) | 1 (1%) | 2 (3%) | 3 (5%) | 1 (2%) | 1 (3%) |
| Vascular disorders, other | 1 (1%) | 1 (1%) | 2 (3%) | 2 (3%) | 1 (2%) | 3 (8%) |
| Vomiting | 0 (0%) | 2 (3%) | 1 (2%) | 0 (0%) | 0 (0%) | 2 (5%) |

Legend Table S1B: Adverse Events (all grades)

Text Table S1B: Data are n (%). The column All grades represents the number of patients with grade 1, 2, and/or 3 TRAEs, counted once per patient regardless of the number of different grades experienced.

| AEs | Grade 1  n = 73 | Grade 2  n = 73 | Grade 3  n = 73 | Grade 4  n = 73 | Grade 5  n = 73 | All grades  n = 73 |
| --- | --- | --- | --- | --- | --- | --- |
| Total | 73 (100%) | 64 (88%) | 24 (33%) | 1 (1%) | 0 (0%) | 73 (100%) |
| Abdominal pain | 40 (55%) | 10 (14%) | 0 (0%) | 0 (0%) | 0 (0%) | 45 (62%) |
| Alopecia | 6 (8%) | 0 (0%) | 0 (0%) | 0 (0%) | 0 (0%) | 6 (8%) |
| Blood creatinine increased | 2 (3%) | 0 (0%) | 0 (0%) | 0 (0%) | 0 (0%) | 2 (3%) |
| Cardiac disorders, other | 6 (8%) | 0 (0%) | 0 (0%) | 0 (0%) | 0 (0%) | 6 (8%) |
| Constipation | 55 (75%) | 13 (18%) | 0 (0%) | 0 (0%) | 0 (0%) | 60 (82%) |
| Decreased appetite | 34 (47%) | 10 (14%) | 1 (1%) | 0 (0%) | 0 (0%) | 36 (49%) |
| Diarrhoea | 31 (42%) | 16 (22%) | 2 (3%) | 0 (0%) | 0 (0%) | 38 (52%) |
| Dizziness | 37 (51%) | 7 (10%) | 0 (0%) | 0 (0%) | 0 (0%) | 40 (55%) |
| Dyspepsia | 46 (63%) | 14 (19%) | 0 (0%) | 0 (0%) | 0 (0%) | 50 (68%) |
| Eye disorders, other | 10 (14%) | 0 (0%) | 0 (0%) | 0 (0%) | 0 (0%) | 10 (14%) |
| Fatigue | 67 (92%) | 27 (37%) | 6 (8%) | 0 (0%) | 0 (0%) | 69 (95%) |
| Flatulence | 54 (74%) | 3 (4%) | 0 (0%) | 0 (0%) | 0 (0%) | 54 (74%) |
| Gastrointestinal disorders, other | 4 (5%) | 0 (0%) | 1 (1%) | 0 (0%) | 0 (0%) | 5 (7%) |
| General disorders, other | 4 (5%) | 3 (4%) | 0 (0%) | 0 (0%) | 0 (0%) | 6 (8%) |
| Headache | 33 (45%) | 3 (4%) | 0 (0%) | 0 (0%) | 0 (0%) | 35 (48%) |
| Hematologic disorders, other | 7 (10%) | 3 (4%) | 1 (1%) | 0 (0%) | 0 (0%) | 9 (12%) |
| Hiccups | 10 (14%) | 0 (0%) | 0 (0%) | 0 (0%) | 0 (0%) | 10 (14%) |
| Infectious disorders, other | 6 (8%) | 3 (4%) | 0 (0%) | 0 (0%) | 0 (0%) | 8 (11%) |
| Insomnia | 40 (55%) | 17 (23%) | 3 (4%) | 0 (0%) | 0 (0%) | 46 (63%) |
| Investigations, other | 8 (11%) | 5 (7%) | 1 (1%) | 0 (0%) | 0 (0%) | 11 (15%) |
| Liver transaminases increased | 14 (19%) | 1 (1%) | 1 (1%) | 0 (0%) | 0 (0%) | 14 (19%) |
| Muscular disorders, other | 13 (18%) | 4 (5%) | 1 (1%) | 0 (0%) | 0 (0%) | 16 (22%) |
| Nausea | 41 (56%) | 14 (19%) | 0 (0%) | 0 (0%) | 0 (0%) | 44 (60%) |
| Nervous disorders, other | 4 (5%) | 0 (0%) | 0 (0%) | 0 (0%) | 0 (0%) | 4 (5%) |
| Neutropenia | 2 (3%) | 1 (1%) | 6 (8%) | 1 (1%) | 0 (0%) | 10 (14%) |
| Palmar plantar erythrodysesthesia | 9 (12%) | 1 (1%) | 0 (0%) | 0 (0%) | 0 (0%) | 10 (14%) |
| Psychological disorders, other | 1 (1%) | 1 (1%) | 0 (0%) | 0 (0%) | 0 (0%) | 2 (3%) |
| Respiratory disorders, other | 8 (11%) | 0 (0%) | 1 (1%) | 0 (0%) | 0 (0%) | 8 (11%) |
| Sensory neuropathy | 25 (34%) | 5 (7%) | 0 (0%) | 0 (0%) | 0 (0%) | 28 (38%) |
| Skin disorders, other | 8 (11%) | 2 (3%) | 1 (1%) | 0 (0%) | 0 (0%) | 8 (11%) |
| Urticaria | 6 (8%) | 2 (3%) | 1 (1%) | 0 (0%) | 0 (0%) | 9 (12%) |
| Vascular disorders, other | 2 (3%) | 2 (3%) | 0 (0%) | 0 (0%) | 0 (0%) | 4 (5%) |
| Vomiting | 2 (3%) | 1 (1%) | 1 (1%) | 0 (0%) | 0 (0%) | 4 (5%) |

## Table S2

Legend Table S2: Summary of rescue medication use for each cycle.

Text Table S2: The total number of rescue medications may exceed the number of patients using rescue medication per cycle, as some patients may have used more than one type of rescue medication on the same day or within the same cycle.

|  | Cycle 1  n = 73 | Cycle 2  n = 66 | Cycle 3  n = 59 | Cycle 4  n = 52 | Cycle 5  n = 37 |
| --- | --- | --- | --- | --- | --- |
| Total (patients) | 16 | 17 | 16 | 14 | 15 |
| CBD oil 5% | - | - | - | 1 | 1 |
| Dexamethasone | 1 | - | 1 | 1 | - |
| Domperidone | 15 | 15 | 16 | 12 | 14 |
| Lorazepam | - | 1 | - | - | 1 |
| Metopimazine | - | 1 | - | - | 1 |
| Olanzapine | - | - | - | - | 1 |
| Ondansetron | 1 | - | - | 1 | 1 |
| Unknown | - | 1 | - | - | - |
